# Upregulation of Mitochondrial Redox Sensitive Proteins in LPS-Treated Stefin B-Deficient Macrophages

**DOI:** 10.3390/cells8121476

**Published:** 2019-11-21

**Authors:** Mojca Trstenjak Prebanda, Janja Završnik, Boris Turk, Nataša Kopitar Jerala

**Affiliations:** 1 Department of Biochemistry, Molecular and Structural Biology, Jožef Stefan Institute, SI-1000 Ljubljana, Slovenia; mojca.prebanda@ijs.si (M.T.P.); janja.zavrsnik@gmail.com (J.Z.); boris.turk@ijs.si (B.T.); 2 International Postgraduate School Jožef Stefan, Jamova 39, SI-1000 Ljubljana, Slovenia; 3 Faculty of Chemistry and Chemical Technology, University of Ljubljana, Večna pot 113, SI-1000 Ljubljana, Slovenia

**Keywords:** cystatin, inflammation, LPS-induced oxidative stress, peroxiredoxin, sulfiredoxin, superoxide dismutases, thioredoxin

## Abstract

Stefin B (cystatin B) is an intracellular inhibitor of cysteine cathepsins and mutations in the stefin B gene, resulting in the development of Unverricht–Lundborg disease, which is a form of myoclonic epilepsy. It was suggested that a key mechanism behind stefin B-mediated disease progression was impaired redox homeostasis. Stefin B-deficient mice were found more sensitive to lipopolysaccharide (LPS)-induced sepsis as a consequence of increased expression of caspase-11 and Nucleotide-binding oligomerization domain, Leucine rich Repeat and Pyrin domain containing (NLRP nflammasome activation and higher levels of mitochondrial reactive oxygen species (ROS). In the present study, we investigated if LPS-triggered oxidative stress affected the protein levels and redox status of redox sensitive proteins—thioredoxin, peroxiredoxins, and superoxide dismutases in macrophages and spleens of LPS-injected mice. LPS challenge was found to result in a marked elevation in mitochondrial peroxiredoxin 3 (Prx3), sulfiredoxin, and superoxide dismutase 2 (Sod2) in stefin B-deficient macrophages and spleens. We determined that sulfiredoxin is targeted to mitochondria after LPS challenge. In conclusion, the upregulation of mitochondrial redox-sensitive proteins Prx3 and Sod2 in stefin B-deficient cells implies a protective role of stefin B in mitochondrial function.

## 1. Introduction

Several recent reports have demonstrated that the cysteine cathepsins and their inhibitors- cystatins play an important role in immune response [1,2]. Stefin B, an endogenous inhibitor of cysteine cathepsins from the cystatin superfamily, is localized in the cytosol, mitochondria, and in the nucleus [3]. Its expression is upregulated upon macrophage activation and oxidative stress [4,5]. Loss-of-function mutations in the stefin B gene result in Unverricht Lundborg disease, a form of myoclonic epilepsy (EPM1) [6,7,8]. Characteristics of the disease are cerebellar granule neuron apoptosis, progressive ataxia, and myoclonic epilepsy [9]. It was shown that the early microglial activation preceded neuronal loss in the brain of stefin B-deficient mice [10]. In addition, as the mice age, more classically activated microglia and increased expression of inducible nitric oxide synthase iNOS and chemokines were determined in stefin B-deficient mice when compared to control animals [11]. Moreover, Lehtinen et al. demonstrated that stefin B-deficiency sensitizes cerebellar granule neurons to oxidative stress-induced cell death [5]. In our past study, we demonstrated that the mice lacking stefin B were significantly more sensitive to lethal lipopolysaccharide (LPS)-induced sepsis, secreted higher amounts of pro-inflammatory cytokines in serum, and have increased NLRP3 inflammasome activation when compared with control animals [12]. We showed that upon LPS stimulation, stefin B was translocated into mitochondria and protected mitochondrial integrity. In addition, we determined increased destabilization of mitochondrial membrane potential and mitochondrial superoxide generation in stefin B-deficient macrophages after LPS challenge [12].

The thioredoxin (Trx) system, composed of NADPH, thioredoxin reductase (TrxR), and Trx, is a key antioxidant system that protects cells from oxidative stress through its disulfide reductase activity [13]. Thioredoxin (Trx) is mainly found in the cytoplasm and is expressed in different cells and tissues where it controls cellular reactive oxygen species (ROS) by reducing the disulfides into thiol groups [14]. Thioredoxin reductase is required for Trx regeneration [15]. Peroxiredoxins have an important role in peroxide detoxification and regulation of hydrogen peroxide signaling is performed by peroxiredoxins (Prx) [16]. The six mammalian Prx isoforms identified to date can be divided into three subgroups that have been designated 2-Cys, atypical 2-Cys, and 1-Cys. The 2-Cys proteins, which include Prx 1, 2, 3, and 4, contain an additional conserved cysteine in the C-terminal region, whereas members of the atypical 2-Cys (Prx 5) and 1-Cys (Prx 6) subgroups do not [17]. Among 2-Cys Prx isoforms, Prx1 and Prx2 are localized in the cytosol, whereas Prx3 is localized exclusively in the mitochondria and its function is to maintain the mitochondrial redox state [16,17,18]. In the cells, cysteine residues of 2-Cys Prxs undergo reversible oxidation to sulfinic acid (Cys-SO_2_H), which in turn could be reversed by the sulfinic acid reductase sulfiredoxin (Srx) [19,20]. Srx catalyzes the ATP-driven rescue of Cys-sulfinic acid derivatives of Prx back to reduced form [21,22]. Srx is present only in eukaryotes and it was first described in yeast [21]. It was demonstrated that Srx translocates from the cytosol to mitochondria in response to hydrogen peroxide-induced oxidative stress and plays a crucial role in the reactivation of sulfinic mitochondrial Prx3 [23,24]. Previous studies have demonstrated that Prx enzymes were up-regulated and exerted a protective antioxidant role in macrophages exposed to LPS [25,26]. Mice lacking Srx and Prx have decreased tolerance to LPS induced sepsis [27,28,29]. In addition, superoxide dismutases are antioxidant enzymes that dismutate superoxide anions to form hydrogen peroxide and molecular oxygen [30]. Cu/Zn superoxide dismutase (Sod1) is found in the cytoplasm, nucleus, and peroxisomes of all mammalian cells where it functions as superoxide scavenger. Sod1 was reported to regulate caspase-1 activaton and endotoxic shock; Sod1-deficient mice were less susceptible to LPS induced endotoxemia [31]. The MnSod, Sod2, is a homotetrameric protein that is found exclusively in mitochondria of mammalian cells [30]. In the mitochondrial matrix, MnSod rapidly scavenges and dismutates superoxide anions into hydrogen peroxide and molecular oxygen [30,32].

The antioxidant response in cells lacking stefin B in LPS-induced oxidative stress is poorly understood. The objective of this study was to determine the effect of stefin B in macrophage intracellular signaling on oxidation of redox sensitive proteins cytosolic and mitochondrial Prx, Srx, Trx, TrxR, Sod1, and Sod2. In the present study we determined the levels of antioxidant proteins in bone marrow-derived macrophages (BMDMs) and spleens of stefin B-deficient mice upon LPS challenge.

## 2. Materials and Methods

### 2.1. Reagents

LPS from *Escherichia coli* (*E. coli*, Sigma, St. Louis, MO, USA) was used at a final concentration of 100 ng/mL for the cellular studies. Antibodies used in western blotting were purchased from Abcam (Cambridge, UK); anti peroxiredoxin 2 (ab109367), anti peroxiredoxin 3 (ab73349), anti peroxiredoxin-SO3, (ab16830), anti sulfiredoxin, (ab92298), antisuperoxide dismutase 1 (SOD1), and Abcam (ab16831) were from Cell Signaling Technology; and anti SOD2 (CY-13141T) and anti β-actin antibodies were from Sigma (A1978).

### 2.2. Animals

Stefin B (cystatin B)-deficient mice were created as described previously [12] and were provided by Dr. R.M. Myers, Stanford University, and bred in our local colony. Mice (8–12 weeks of age) used in this study were wild type (WT) and Stefin B-deficient (KO), fully backcrossed to FVB/N background. Maintenance and breeding of the animals used in this study were performed in accordance with Slovene law for animal protection, as published in May 2013.

All animal studies were conducted in accordance with the Administration of the Republic of Slovenia for food safety, veterinary, and plant protection. Procedures for animal care and experiments were in accordance with the “Guide for the Care and Use in Laboratory Animals”, approved by the Veterinary Administration of the Republic of Slovenia and the government Ethical Committee (U34401-49/2014/7).

### 2.3. Stimulations and Systemic LPS Challenge In Vivo

Mouse primary bone marrow-derived macrophages (BMDMs) were obtained by ex vivo differentiation from mouse bone marrow progenitors in the presence of L929 conditioned medium over 7 days, as described previously [12]. Cells were kept in DMEM supplemented with 20% FBS, 1% penicillin/streptomycin, and 2 mM l-glutamine. BMDMs were seeded in 6-well plates (10^6^ cells/well) for lysate preparation. Cells were primed with LPS (100 ng/mL) for 4 or 24 h. For the spleen whole lysate preparations, stefin B KO and WT mice were intraperitoneally injected with LPS at 3 mg/kg (*E. coli* 055:B5, Sigma) and sacrificed 4 h after injection.

### 2.4. Cell Lysate Preparation and Western Blot Analysis

Monolayer BMDMs at 80% confluence were first washed with ice-cold PBS, and cell lysates were prepared as described previously [12]. Mouse spleen tissue lysate was prepared by homogenization in the Nonidet P-40 lysis buffer (20 mM Tris–HCl (pH 7.4), 150 mM NaCl, 1 mM EDTA, 1% Nonidet P-40, 10% glycerol, 1 mM sodium orthovanadate, 10 mM NaF, 10 mM β-glycerophosphate), supplemented with the complete protease inhibitor cocktail (Sigma) and the phosphatase inhibitor cocktail (Sigma, St. Louis, MO, USA). Tissue and cell debris were removed by centrifugation. Protein concentration was determined with Bradford reagent (Bio-Rad, Muenchen, Germany). The lysates were subjected to electrophoresis on 12.5% SDS-polyacrylamide gels followed by western blotting, as described previously [12]. Protein bands were visualized with ECL (Amersham Biosciences, Amersham, UK) according to the manufacturer’s instructions. The signals were quantified by densitometry analysis using ImageJ software (ImageJ 1.01, NIH, MD, USA) (http://rsb.info.nih.gov/ij/index.html), according to the instructions, as described (https://www.unige.ch/medecine/bioimaging/files/2014/1208/6025/GelAnalysis).

### 2.5. Confocal Microscopy

WT BMDMs were seeded on coverslips, and were left untreated or stimulated with LPS. After the indicated time of the treatment, cells were washed, probed with MitoTracker Red CMXRos (100 nm) in OptiMEM for 45 min at 37 °C, then fixed with 4% paraformaldehyde in PBS, pH 7.2, for 15 min, and permeabilized with 0.1% Triton X-100 for 10 min in PBS. Nonspecific staining was blocked with 3% BSA (Sigma-Aldrich) in PBS, pH 7.4, for 40 min. Sulfiredoxin was labeled with goat anti-sulfiredoxin antibodies. Highly cross-adsorbed donkey anti-goat IgG antibodies, labeled with Alexa Fluor 488 and obtained from Life Technologies-Molecular Probes, were used as secondary antibodies. After the final wash, cells were mounted on slides with Prolong Gold Antifade Mountant containing 4′,6′-diamidino-2-phenylindole (DAPI) (Thermo Scientific, Invitrogen, Carlsbad, CA, USA). Control samples were run in the absence of primary antibodies. Immunofluorescence microscopy of optical sections was performed by using a confocal laser scanning microscope Leica TCS SP5 X (Leica MicroSystems, Wetzlar, Germany). The fluorophores were excited with selected lines from a tunable white light laser (460–670 nm) or a diode laser (405 nm). In order to minimize crosstalk of fluorophores, sequential scanning was performed. Leica Application Suite Advanced Fluorescence software (LAS AF, version 2.7.3.9723, Leica MicroSystems, Wetzlar, Germany) was used for the image analysis. Quantitative colocalization analysis was performed and Pearson correlation coefficient calculated using Leica software.

### 2.6. Statistical Analysis

Statistical significance of the results was determined using unpaired Student’s *t*-test, assuming unequal variances. All data are presented as the mean ± standard error of the mean. Significance was defined as *p* < 0.05.

## 3. Results

### 3.1. Thioredoxin, Thioredoxin Reductase, and Peroxiredoxin Proten Levels in LPS-Stimulated BMDMs and Stefin B-Deficient BMDMs

**Figure 1:**
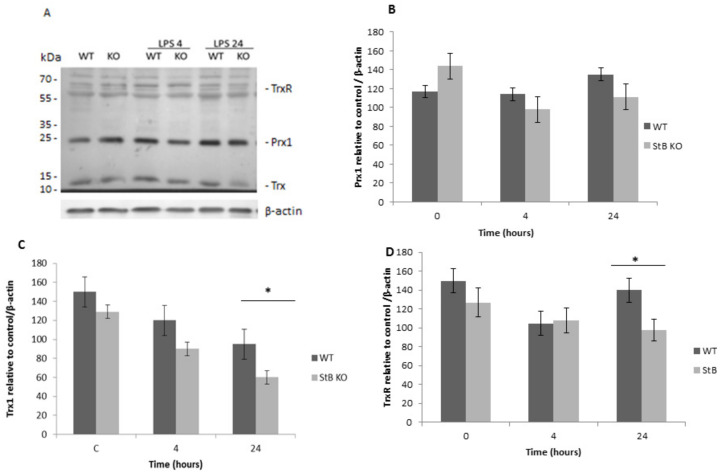
Effects of lipopolysaccharide (LPS) treatment on thioredoxin reductase 1 (TrxR1), peroxiredoxin 1 (Prx 1), and thioredoxin 1 (Trx1) protein levels in wild type (WT) and stefin B deficient (KO) bone marrow-derived macrophages (BMDMs). Western blot analysis (**A**) was used to examine levels of Prx1, (**B**) Trx1, (**C**) and TRxR1 (**D**) in BMDMs from wild typeWT) and stefin B-deficient (KO) mice and β-actin controls. BMDMs were treated with 100ng/mL LPS for 4 and 24 h and expression was analyzed with western blots with antibodies specific for the PRX pathway (ab184868). Representative blots depict typical immunoblots from three different biological samples. Statistically significant differences versus control group, * *p* < 0.05.

First, we examined the protein levels of Prx1, which functions in the cytosol as a regulator of hydrogen signaling through its peroxidase activity [17]. We aimed to investigate if the lack of stefin B influenced Prx1 protein levels in WT and stefin B KO BMDMs after LPS challenge. Therefore, we examined not only Prx1, but also the other enzymes involved in the Prx-related signaling pathways—thioredoxin 1 (Trx1) and thioredoxin reductase (TrxR)—which can hamper the regeneration rate of Prx. In the control (unstimulated cells), Prx1 protein levels were upregulated in unstimulated stefin B KO BMDMs, but the differences were not statistically significant. In LPS-stimulated BMDMs, there were no significant differences between the two phenotypes, WT BMDMs and stefin B KO BMDMs (Figure 1). In BMDMs, LPS initiated toll like receptor 4 TLR4 signaling and translocation of the pro-inflammatory transcription factor nuclear factor kappa-light-chain enhancer of activated B cells (NF-κB) into the nucleus from the cytoplasm to induce gene transcription [33]. In WT and stefin B KO BMDMs, we confirmed translocation of p65 subunit of NF-κB into the nucleus 40 min after LPS treatment Figure S1), confirming that LPS treatment was effective. When we compared the protein levels in WT and stefin B KO BMDMs 4 and 24 h after LPS stimulation, we could not confirm any significant differences between the phenotypes (Figure 1B). However, the protein levels of Trx1 were lower in untreated stefin B KO BMDMs, but only in BMDMs after 24 h stimulation with LPS; the differences were significantly lower in KO BMDMs when compared to WT BMDMs (Figure 1C). In LPS-stimulated stefin B KO and WT BMDMs, no significant differences between the phenotypes were detected 4 h after LPS stimulation in TrxR protein levels (Figure 1D). Twenty-four hours after LPS challenge, the levels of TrxR were lower in stefin B KO BMDMs (Figure 1D). We concluded that stefin B deficiency did not have a major influence on Prx1 protein levels in the BMDMs, whereas the lower levels of Trx1 could contribute to higher sensitivity of stefin B KO mice to LPS-induced sepsis. In addition, we confirmed that the BMDMs and spleens prepared from stefin B deficient mice do not express stefin B protein (Figure S2).

### 3.2. Elevated Hyperoxidized Prx2 and Prx3 in LPS Challenged Stefin B-Deficient BMDMs

**Figure 2:**
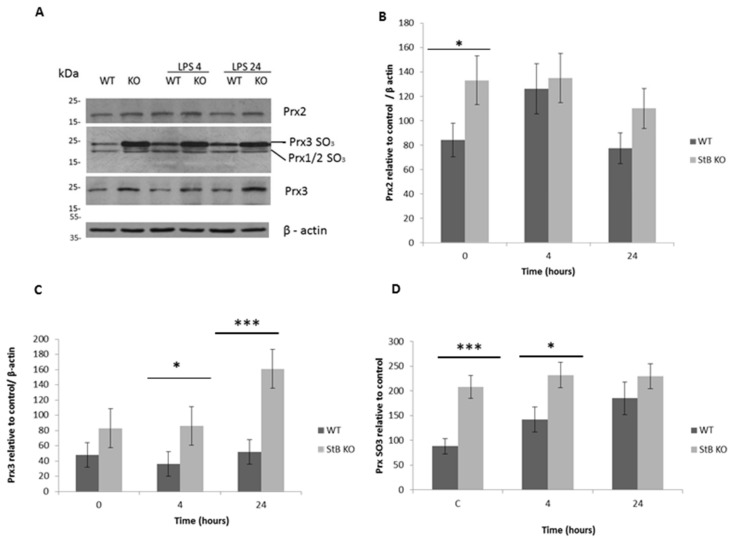
Prx3 was upregulated in stefin B KO BMDMs. Western blots of Prx2, Prx3, and Prx-SO_3_ (**A**). Protein levels of Prx2 (**B**), Prx3 (**C**), Prx-SO_3_ (**D**), and β-actin in whole BMDMs cell lysates of mice with the wild type FVB (WT) and stefin B deficient (KO) mice. BMDMs were treated with 100ng/mL LPS for 4 and 24 h and protein levels were determined with antibodies specific to PrxSO_3_, Prx2, or Prx3 in order to confirm the band location of each protein. The western blots are representative of three independent experiments. Statistically significant differences versus control group, * *p* < 0.05; ** *p* < 0.01.

Next, we examined whether stefin B deficiency affected the expression and oxidation state of cytosolic Prx2 and mitochondrial Prx3 in BMDMs of WT and stefin B KO mice after LPS stimulation (Figure 2A). Previously, it was reported that Prx3 deficiency sensitizes macrophages to LPS-induced oxidative stress [25]. We performed western blot analysis and used antibodies to hyperoxidise Prx-(Prx-SO_3_) and determine the oxidative state of Prx2 (22kDa) and Prx3 (25kDa) in LPS-challenged and in control BMDMs from WT and KO mice. The monomeric forms of both Prx1 and Prx2 could be detected at 22 kDa and using antibodies for peroxiredoxin-SO3, and we labeled them Prx1/2 SO_3_, whereas Prx 3 monomer was detected at 25 kDa, and it was labeled as Prx3 SO_3_. The protein levels of Prx2 were upregulated in stefin B KO BMDMs, but 4 h after LPS stimulation the protein levels of Prx2 were comparable in WT and stefin B KO BMDMs (Figure 2B). Twenty-four hours after LPS challenge, stefin B KO BMDMs had higher levels of Prx2 than WT BMDMs, and the differences between the phenotypes were not significant (Figure 2B). Upon LPS stimulation, the protein levels of Prx3 were upregulated in both WT and stefin B KO BMDMs (Figure 2C), and the levels of hyperoxidized Prx2 and Prx3 were higher in untreated stefin B KO BMDMS 4 h after LPS challenge (Figure 2D). Stefin B KO BMDMs had higher levels of hyproxidized Prx3, even in control BMDMs (Figure 2D). The levels of hyperoxidized Prx2 were higher in stefin B KO BMDMS before the LPS stimulation (Figure 2D). In LPS-stimulated BMDMs, we determined increased levels of Prx3-SO_3_ in WT and stefin B KO BMDMS, 4 and 24 h after LPS stimulation (Figure 2D). The hyperoxidized form with a weight of 25 kDa was identical to a mitochondrial isoform of Prx3. These results indicated that a stefin B-deficiency disturbed the Prx redox system mostly by hyperoxidation of mitochondrial Prx3 in LPS-stimulated stefin B KO BMDMs.

### 3.3. Upregulation of Srx in LPS-Stimulated BMDMs from Stefin B Deficient Mice

**Figure 3:**
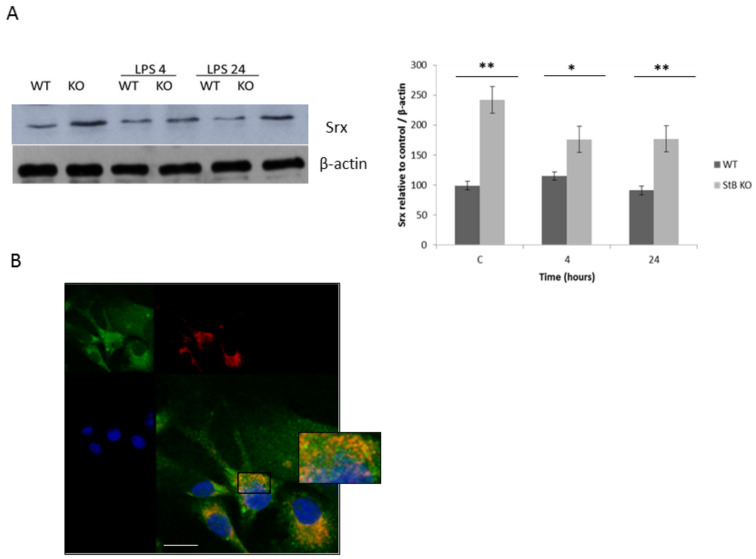
Sulfiredoxin was up-regulated in LPS treated BMDMs from stefin B-deficient mice and targeted into mitochondria upon LPS treatment. (**A**) Mouse BMDMs were stimulated with LPS (100 ng/mL) for 4 and 24 h. Srx protein levels were determined by western blotting of total cell lysates and compared to controls, as described in Section 2. WT BMDMs were treated with LPS (100 ng/mL) for 24 h. The western blots are representative of three independent experiments. Statistically significant differences versus control group, * *p* < 0.05; ** *p* < 0.01. (**B**) Mitochondria (red) were labeled with MitoTracker Red CMXRos (Ex/Em 579⁄599 nm) fixed as described in Section 2, stained with anti-sulfiredoxin antibodies (green), and labeled with Alexa conjugated secondary antibodies (Alexa 488). Nuclei were stained with 4′,6′-diamidino-2-phenylindole (DAPI). The images were merged to visualize co-localization of sulfiredoxin and mitochondria. The image is representative of three independent experiments. Scale bar, 10 μm.

It was reported that the hyperoxidized Prx3 could be reduced to the catalytically active thiol form by sulfiredoxin [34]. Therefore, we asked the question if protein levels of Srx are upregulated in stefin B KO BMDM. Srx has been demonstrated as the sole enzyme responsible for the reactivation of 2-Cys peroxiredoxins, Prx2, and Prx3. In stefin B KO BMDMs, we determined increased expression of Srx in unstimulated BMDMs and in LPS-stimulated macrophages (Figure 3A). Although Srx is predominantly found in cytosol, it was demonstrated that upon hydrogen peroxide-induced oxidative stress that it could be translocated into mitochondria [23]. Next, we asked if Srx is targeted to mitochondria upon LPS stimulation. The co-localization between Mitotracker Red CMXRos and Srx was quantified by the Pearsons correlation coefficient (PCC) using Leica software. The calculated PCC for the whole image was PCC = 0.635 ± 0.118 and PCC = 0.59 ± 0.11 for the region of interest, marked in Figure 3B, indicating partial co-localization. Although Srx was found also in mitochondria of stefin B KO BMDMs, we determined increased Prx3 hyperoxiation, suggesting that the amount of Srx was not sufficient to compensate for the increased oxidative stress and Prx3 hyperoxidation in stefin B KO BMDMs.

### 3.4. Protein Levels of Sod1 in WT and KO BMDMs and Spleens

**Figure 4:**
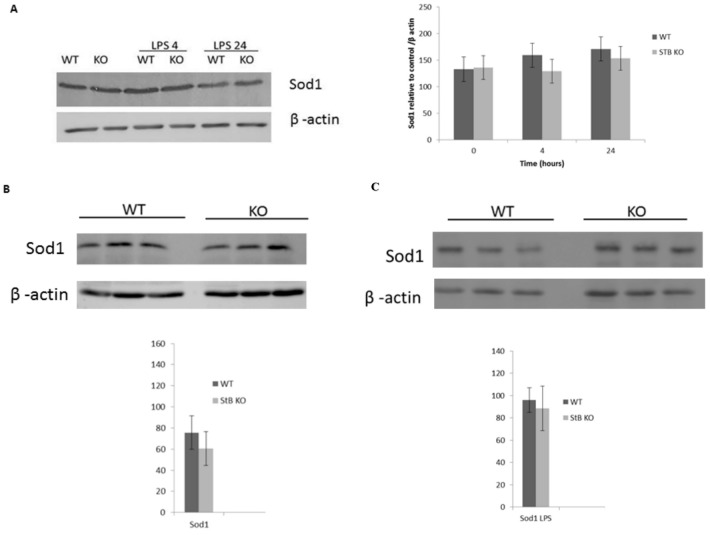
No difference in superoxide dismutase (Sod1) protein levels in LPS-treated macrophages and spleens from stefin B-deficient mice. Mouse BMDMs were stimulated with LPS (100 ng/mL) for 4 and 24 h. Sod1 protein levels were determined by western blotting of total cell lysates, as described in Section 2. (**A**) Age-matched control FVB/N mice (WT) and stefin B-deficient (KO) mice were left untreated or injected with LPS (3 mg/kg body weight); 4 h after injection, the mice were sacrificed and spleens were removed and tissue lysates were prepared, as described. Spleen lysates of untreated animals (**B**) or LPS-challenged animals (**C**) were separated by SDS–PAGE and analyzed by western blots with Sod1-specific antibodies and β-actin controls. The western blots are representative of three independent experiments.

We hypothesized that Sod1 upregulation may compensate for the lack of stefin B in LPS-challenged BMDMs and spleens. First, we examined the protein levels of Sod1 in WT and stefin B KO BMDMs; however, we did not observe any significant differences between WT BMDMs and KO BMDMs in Sod1 protein levels, between unstimulated BMDMs, or after LPS treatment (Figure 4A). Also, when we analyzed spleens lysates of LPS-challenged WT and stefin B KO mice with western blots, we did not determine any differences, nor was this the case in spleen lysates from LPS challenged stefin B KO mice, nor in cell lysates from WT LPS treated spleens (Figure 4B,C). We concluded that stefin B deficiency did not result in Sod1 upregulation.

### 3.5. Sod 2 is Upregulated in Stefin B-Deficient BMDMs and Spleens

**Figure 5:**
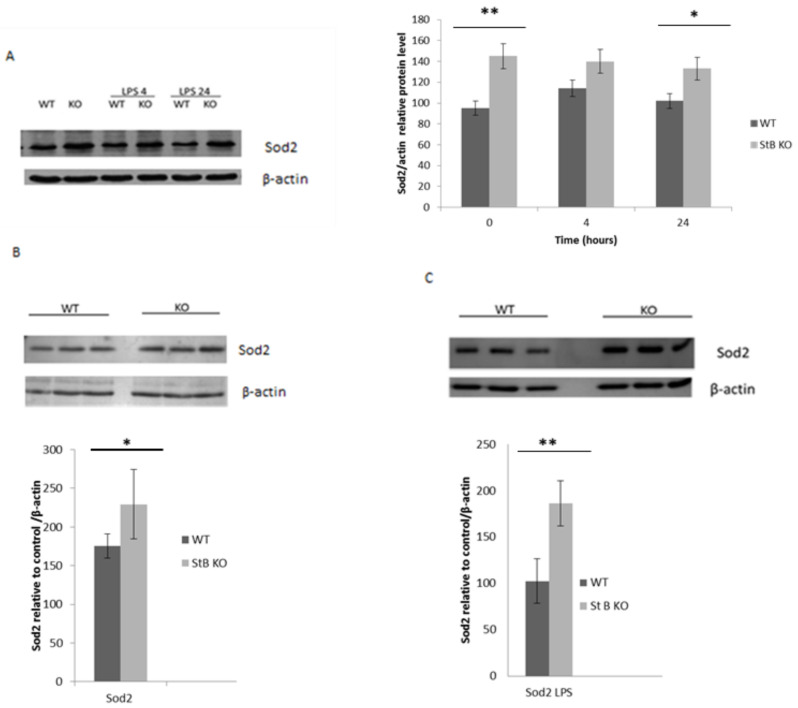
Sod2 was up-regulated in LPS treated macrophages and spleens from stefin B-deficient mice. (**A**) Mouse BMDMs were stimulated with LPS (100 ng/mL) for 4 and 24 h. Sod2 protein levels were determined by western blotting of total cell lysates with antibodies against Sod2 and compared to β-actin controls, as described (**A**) Age-matched control FVB/N mice (WT) and stefin B KO (KO) were left untreated or were injected with LPS (3 mg/kg body weight). Then, 4 h after injection, the mice were sacrificed and spleens were removed. Spleen lysates of untreated animals (**B**) or LPS-challenged animals (**C**) were analyzed by western blots with Sod2-specific antibodies and β-actin antibodies as loading controls. The western blots are representative of three independent experiments. Statistically significant differences versus control group, * *p* < 0.05; ** *p* < 0.01.

Sod2 (Mn-Sod2) is localized within mitochondria and it efficiently eliminates the superoxide generated in the mitochondrial respiratory chain [30]. Even in untreated stefin B KO BMDMs, Sod2 protein levels were higher than in WT control cells (Figure 5A). In our experiments, we determined significantly higher Sod2 protein levels in stefin B KO BMDMs than in WT BMDMs 24 h after LPS stimulation (Figure 5A) with western blot analysis. In spleens from stefin B KO mice injected with LPS, higher levels of Sod2 were determined in comparison to control animals. We propose that the higher levels of Sod2 in stefin B KO BMDMs compensated for increased mitochondrial ROS previously reported in LPS-challenged stefin B KO BMDMs.

## 4. Discussion

Stefin B deficiency was shown to be implicated in impaired redox homeostasis, resulting in an increased oxidative stress-induced cell death [5,35]. In stefin B-deficient mouse model, it was demonstrated that the pathogenesis of the EPM1 disease is a consequence of increased oxidative stress and inflammation in the cerebellum, as well as in the periphery of the organism [11,36]. In this study, we examined the levels of redox sensitive proteins Prx and Trx in stefin B-deficient mice upon LPS challenge.

The protective role of Prxs in inflammation was revealed by the higher sensitivity of Prx–deficient mice to LPS-induced sepsis [27,29]. Sun et al. reported that the mice deficient in Prx1 were significantly more sensitive to LPS-induced sepsis and had lower serum levels of interleukin 10 IL-10 [29]. Interestingly, we demonstrated earlier that BMDMs prepared from stefin B-deficient mice secreted lower amounts of IL-10 after LPS challenge [4]. Therefore, we hypothesized that the downregulation of Prx1 may contribute to the sensitivity to LPS in stefin B-deficient mice. However, we did not determine any significant differences between the phenotypes in the protein levels of Prx1 in BMDMs after LPS challenge, indicating that the Prx1 does not contribute to the phenotype observed in stefin B KO mice. We also examined the levels of Trx and TrxR, which can hamper the regeneration rate of Prx in stefin B KO and WT BMDMs upon LPS stimulation. However, the protein levels of Trx were lower in untreated stefin B KO BMDMs, 4 and 24 h after LPS challenge, when compared to WT BMDMs (Figure 1). Brenner et al. reported elevated plasma levels of Trx in patients with severe sepsis or septic shock [37]. We cannot exclude the possibility that the lower protein levels of Trx1 in stefin B KO BMDMs are a consequence of the increased secretion from the cells after LPS challenge. In the protein levels of TrxR, we did not confirm any differences between stefin B KO and WT BMDMs (Figure 1).

Prx2 is an important antioxidant enzyme because of its great abundance in cells and its high reaction rate with hydrogen peroxide [38]. Similar to Prx2-deficient BMDMs, in stefin B KO BMDMs we determined significantly higher levels of NO and better Nuclear factor NF-kappa B signaling [4,12]. For the mouse macrophages challenged with LPS, NF-κB-binding element was reported to be necessary and sufficient to initiate transcription of the iNOS gene [39]. In our previous work, we reported that stefin B-deficient BMDMs secrete significantly higher levels of NO compared to WT BMDMs and have increased iNOS mRNA expression when compared to control cells [4]. In addition, in reporter macrophage cell line RAW-blue, (Invivogene, San Diego, CA, USA) which overexpressed stefin B, we confirmed a decreased NF-κB activation upon LPS stimulation [12]. However, the precise mechanism by which stefin B influences NF-κB signaling has not been elucidated yet.

The levels of hyperoxidized Prx2 were higher in stefin B KO BMDMS before the LPS stimulation (Figure 2B). Four hours after LPS stimulation, the protein levels of Prx2 were comparable in WT and stefin B KO BMDMs, and after 24 h, LPS challenge stefin B KO BMDMs had higher levels of Prx2; however, the differences were not significant. In Prx2-deficient mice, higher levels of tumor necrosis factor alpha TNF-α, IL-6, and NO in the serum were determined, in comparison to WT control cells [27]. In stefin B-deficient mice, we reported higher levels of TNF-α in the serum 2 h after LPS challenge, but the levels of IL-6 in the serum were comparable between the phenotypes [12]. Regarding the non-significant upregulation of Prx2 in stefin B-deficient BMDMs, we propose that Prx2 does not play a major role in reducing oxidative stress in LPS-stimulated stefin B BMDMs. We determined higher levels of mitochondrial Prx3 in stefin B KO BMDMs, as well as higher Prx3 hyperoxidation (Figure 2C,D). Li et al. reported that Prx3-deficient mice have increased susceptibility to LPS-induced oxidative stress and LPS-induced lung injury [40]. Moreover, it was reported that Prx3-deficient macrophages increased intra cellular ROS production and TNF-α secretion in response to LPS [25]. Prx3 depletion or inactivation decreases mitochondrial membrane potential, leading to mitochondrial dysfunction [41]. The increased Prx3 expression could partially compensate for the increased mitochondrial ROS determined in stefin B KO BMDMS upon LPS challenge. Mitochondrial Srx could regenerate hyperoxidized Prx3. We therefore examined the protein levels and localization of Srx in BMDMs. Kil et al. reported that amounts of Prx3-SO_3_ and Srx undergo circadian oscillation in the mitochondria of specific tissues of mice under normal conditions [24]. Cytosolic Srx was targeted into mitochondria by a mechanism that required formation of a disulfide-linked complex with heat shock protein 90 [24]. We have shown increased Srx localization in the mitochondria upon LPS stimulation. However, the increased levels of Prx3 and Srx in mitochondria could not compensate for the increased mitochondrial superoxide, determined in stefin B KO BMDMs upon LPS challenge [12]. Interestingly, the increased expression of Srx1 and Prx1 was demonstrated in M1-classically activated macrophages [26]. Moreover, it was reported that in the spleens of stefin B-deficient mice that an increased number of classically activated macrophages (M1) is present, in comparison to control animals [36].

In stefin B-deficient BMDMs, we determined increased destabilization of mitochondrial membrane potential and mitochondrial superoxide generation [12]. Superoxide is a reactive molecule but it can be converted to hydrogen peroxide by superoxide dismutase—Sod1 in the cytosol and Sod2 in the mitochondria [30]. Superoxide dismutase Sod1 and stefin B are both encoded on chromosome 21 in humans and are overexpressed in brains of patients with Down syndrome [42]. However, the lack of stefin B did not result in changed protein levels of Sod1. Sod2, MnSOD, is an integral mitochondrial protein known as a first-line antioxidant defense against superoxide radical anions produced as by-products of the electron transport chain. The previous study reported the increased expression of Sod2 in LPS-stimulated macrophages [43]. On our model, we showed not only its upregulation after LPS challenge, but also that stefin B-deficient animals express more Sod2 after LPS stimulation in BMDMs and spleens than control mice.

## 5. Conclusions

During the last decade, several lines of evidence have suggested that not only peroxiredoxins but also cystatins may be important players in innate immune response and inflammation. Specifically, stefin B was demonstrated as protecting macrophages against high levels of ROS produced during inflammatory processes. The main and novel result presented in this study is that the lack of stefin B in BMDMs leads to a significant increase in the expression of the mitochondrial antioxidant proteins Prx3, Srx, and Sod2 in response to LPS challenge. The observed results are in line with our previous report that stefin B KO BMDMs generate higher levels of mitochondrial ROS after LPS stimulation and inflammasome activation [12]. The protective effects of redox-sensitive proteins, peroxiredoxins, and cystatins suggest that they offer a potential for therapy of sepsis and inflammation.

## Supplementary Materials

The supplementary materials are available online at https://www.mdpi.com/2073-4409/8/12/1476/s1.
