# Correction: Trstenjak Prebanda et al. Upregulation of Mitochondrial Redox Sensitive Proteins in LPS-Treated Stefin B-Deficient Macrophages. *Cells* 2019, *8*, 1476

**DOI:** 10.3390/cells13171454

**Published:** 2024-08-30

**Authors:** Mojca Trstenjak Prebanda, Janja Završnik, Boris Turk, Nataša Kopitar Jerala

**Affiliations:** 1 Department of Biochemistry, Molecular and Structural Biology, Jožef Stefan Institute, SI-1000 Ljubljana, Slovenia; mojca.prebanda@ijs.si (M.T.P.); janja.zavrsnik@gmail.com (J.Z.); boris.turk@ijs.si (B.T.); 2 International Postgraduate School Jožef Stefan, Jamova 39, SI-1000 Ljubljana, Slovenia; 3 Faculty of Chemistry and Chemical Technology, University of Ljubljana, Večna pot 113, SI-1000 Ljubljana, Slovenia

## Error in Figure

In the original publication [1], there was a mistake in Figure 4 as published, the western blots of Figure 4A were mistakenly taken from another figure. The corrected Figure 4 appears below.

The authors state that the scientific conclusions are unaffected. This correction was approved by the Academic Editor. The original publication has also been updated.

## Figures and Tables

**Figure 4:**
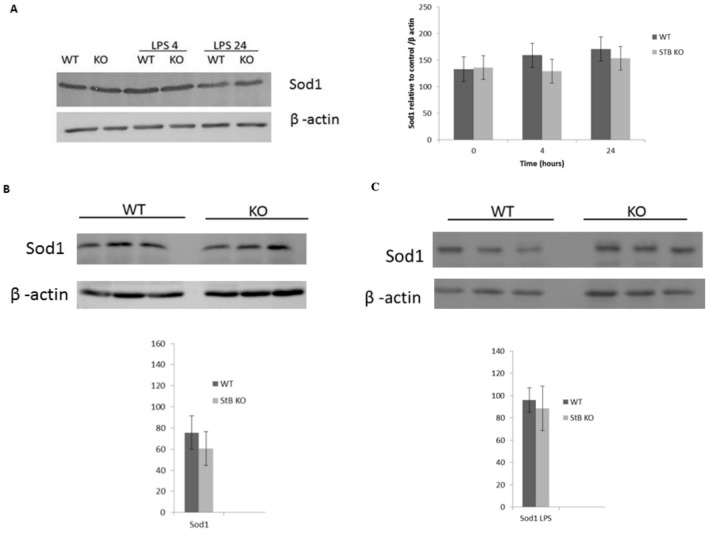
No difference in superoxide dismutase (Sod1) protein levels in LPS-treated macrophages and spleens from stefin B-deficient mice. Mouse BMDMs were stimulated with LPS (100 ng/mL) for 4 and 24 h. Sod1 protein levels were determined by western blotting of total cell lysates, as described in Section 2. (**A**) Age-matched control FVB/N mice (WT) and stefin B-deficient (KO) mice were left untreated or injected with LPS (3 mg/kg body weight); 4 h after injection, the mice were sacrificed and spleens were removed and tissue lysates were prepared, as described. Spleen lysates of untreated animals (**B**) or LPS-challenged animals (**C**) were separated by SDS–PAGE and analyzed by western blots with Sod1-specific antibodies and β-actin controls. The western blots are representative of three independent experiments.
